# Barriers of artificial intelligence implementation in the diagnosis of obstructive sleep apnea

**DOI:** 10.1186/s40463-022-00566-w

**Published:** 2022-04-25

**Authors:** Hannah L. Brennan, Simon D. Kirby

**Affiliations:** Faculty of Medicine, Memorial University of Newfoundland and Labrador, 98 Pearltown Rd, St. John’s, NL A1G 1P3 Canada

**Keywords:** Artificial intelligence, Obstructive sleep apnea, Diagnosis, Barriers

## Abstract

**Background:**

Obstructive sleep apnea is a common clinical condition and has a significant impact on the health of patients if untreated. The current diagnostic gold standard for obstructive sleep apnea is polysomnography, which is labor intensive, requires specialists to utilize, expensive, and has accessibility challenges. There are also challenges with awareness and identification of obstructive sleep apnea in the primary care setting. Artificial intelligence systems offer the opportunity for a new diagnostic approach that addresses the limitations of polysomnography and ultimately benefits patients by streamlining the diagnostic expedition.

**Main body:**

The purpose of this project is to elucidate the barriers that exist in the implementation of artificial intelligence systems into the diagnostic context of obstructive sleep apnea. It is essential to understand these challenges in order to proactively create solutions and establish an efficient adoption of this new technology. The literature regarding the evolution of the diagnosis of obstructive sleep apnea, the role of artificial intelligence in the diagnosis, and the barriers in artificial intelligence implementation was reviewed and analyzed.

**Conclusion:**

The barriers identified were categorized into different themes including technology, data, regulation, human resources, education, and culture. Many of these challenges are ubiquitous across artificial intelligence implementation in any medical diagnostic setting. Future research directions include developing solutions to the barriers presented in this project.

**Graphical abstract:**

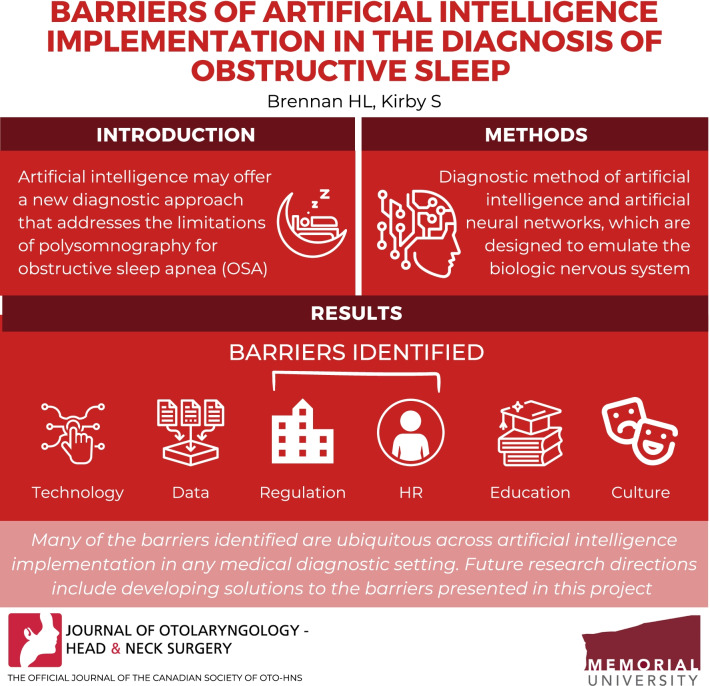

## Background

### The evolution of obstructive sleep apnea diagnosis

Obstructive sleep apnea (OSA) is a common clinical condition, affecting 14% of men and 5% of women, characterized by a cessation of airflow due to upper airway obstruction with simultaneous respiratory effort present [1]. Despite the high prevalence, the number of people diagnosed and seeking treatment is much less, reflecting lack of accessibility in treatment and testing and lack of correlation with the index measured on diagnostic test [2]. Significant untreated OSA is associated with increased risk of cardiovascular disease, metabolic disease, cancer, higher rates of health care use and both traffic and occupational accidents [3–5]. Current awareness and detection of OSA in the primary care setting is a major contributor to the disease process, as it is not routinely screened for and there are limitations to utilizing screening tools, like the STOP-Bang questionnaire, in the general public [2, 6]. Navigating the path from diagnosis to effective treatment is also challenging and requires a multidisciplinary approach and interprofessional collaboration, including sleep specialists, dental sleep medicine and physical therapists [7].

Polysomnography (PSG), categorized as level 1, is the diagnostic gold standard for OSA. PSG utilizes electroencephalogram (EEG), electrooculogram (EOG), electrocardiogram (ECG), pulse oximetry, airflow and respiratory effort to report the Apnea–Hypopnea Index (AHI), which refers to the number of obstructions of airflow events divided by the sleep time [8, 9]. The diagnosis requires an “AHI of 15 or more events per hour or five or more events per hour in the presence of symptoms or cardiovascular comorbidities.” [4] Limitations associated with PSG include a significant cost, both financially and in regards to labor, facilities and specialists required [10]. The limited availability and accessibility challenges with PSG, has generated long wait times and increased disease burden [11].

Home sleep apnea testing (HSAT), categorized as level 3, has been associated with greater patient satisfaction, lower cost and waiting time to diagnosis and subsequent treatment [12]. HSAT is useful in diagnosing patients with a high pretest probability of OSA without unstable comorbidities. However it is not recommended in diagnosing patients with significant comorbid conditions or screening asymptomatic populations, and has been associated with high rates of signal loss and increased study failure [12–14]. The WatchPAT device (Itamar Medical, Ltd) has been an important development in HSAT, in which peripheral arterial tonometry (PAT), pulse oximetry, heart rate and actigraphy data are gathered by the device and analyzed to detect respiratory sleep disturbances [15]. WatchPAT accuracy has been improved by utilizing demographic and anthropometric data [16, 17]. Cardiopulmonary coupling (CPC) is an algorithm derived from the autonomic and respiratory signals recorded by a wearable device, utilizing ECG or photo plethysmography (PPG) and has been shown to have a diagnostic accuracy comparable to PSG; 100% sensitivity and 93.63% specificity [18, 19].

The first-line, gold standard treatment for OSA is nasal continuous positive airway pressure (CPAP), which serves as a pneumatic splint to stabilize the upper airway and is effective when used with appropriate adherence [20, 21]. The factors that influence adherence should be considered in prescribing CPAP, including socio-demographic and psychosocial characteristics, disease severity and side effects [22]. Despite machine improvements and compliance interventions, the non-adherence rates remain significantly high at 30–40% [23]. A study showed how the implications of adherence may challenge CPAP as the gold-standard, and demonstrated that surgery should also be considered in the context of an individual’s anatomical features, OSA severity and treatment goals [24]. This concept of considering individual anatomy guiding treatment is also evident in a study which developed a pediatric OSA algorithm and demonstrated that tonsil size was the strongest predictor of adenotonsillectomy (AT) [25]. Myofunctional therapy (MT) is another treatment option which involves oropharyngeal exercises and has been shown to decrease AHI by 50% in adults [26, 27].

## Methodology

(P) The population focus of this study was adults with obstructive sleep apnea. (I) The diagnostic method of artificial intelligence and artificial neural networks. (C) The use of AI in the diagnosis of OSA was mainly compared to the gold-standard of PSG, but HSAT and other methods were also discussed. (O) The barriers in implementing AI in the diagnosis of OSA were identified in terms of technology/interpretability, culture, human resources, data/biases, regulation, privacy, education. The inclusion criteria analyzed the keywords obstructive sleep apnea and artificial intelligence. Non-English articles were excluded from this review.

## Main body

### The role of AI in obstructive sleep apnea diagnosis

Artificial intelligence (AI) creates an avenue for screening and diagnosing OSA, which can reduce costly PSG use and streamlining path to effective treatment.

AI is being applied to assist healthcare workers with tasks and decisions that require analysis of complex data, such as establishing a diagnosis, predicting outcomes and treatment decisions. There are various AI systems being employed, including artificial neural networks (ANNs), fuzzy expert systems, evolutionary computation and hybrid intelligent systems [28]. The most popular AI technique employed within medicine is ANNs, which are computer analytic programs designed to emulate the intricacy, structure and behavior of the biological nervous system [28]. ANNs are composed of layers of processing elements or ‘neurons’ which are connected in a network. Each processing element in the network has weighted inputs, transfer function and one output [29]. The weights or coefficients of the inputs are adjustable parameters and the transfer function allows for non-linearity [29]. The network is able to ‘learn’ during training by exposure to data sets and their outcomes, through subsequent adjustments or optimizations to the connections between the neurons [29]. Following training, the ANN can be tested by inputting new unseen data and evaluating the predicted outcome. AI is currently being employed in various medical settings, including detecting ischemia in myocardial perfusion imaging, pulmonary embolism diagnosis, and classification of lung cancer with computerized tomography (CT) images [30–32].

One of the first studies to examine the use of AI in the diagnosis of OSA focused on a generalized regression neural network (GRNN), trained with 23 clinical variables [33]. This neural network demonstrated the ability to accurately rule in OSA from clinical data, with a sensitivity and specificity of 98.9% and 80% respectively [33]. This study introduced how neural networks could potentially determine whether PSG was required in non-OSA patients and could streamline OSA patients towards a therapeutic study rather than a diagnostic study [33]. Another early study developed an ANN with 12 input variables that predicted AHI, which allowed for prediction of diagnosis as well as severity, rather than a binary OSA or non-OSA diagnosis [34]. AHI is only one consideration and is limited in its ability to predict adverse effects and responsiveness to treatment [35]. AI offers an opportunity to consider more factors beyond AHI, which can provide more information about severity, OSA endophenotypes and can inform treatment selection [36, 37].

Since OSA is diagnosed based on clinical features, another ANN was developed focusing on 4 inputs that are easily accessible: sex, age, body mass index (BMI), and snoring status [38]. The simplified input data employed in this neural network reflected how ANN can allow for accurate prediction of OSA in the office setting without need for other resources like oximetry or PSG. The ANN was assessed with the multilayer perceptron (MLP) network and demonstrated a diagnostic accuracy of 86.6% [38]. Similarly, another study developed a prediction model based on an AI system to detect sleep apnea from easily obtained information in a questionnaire without any biochemical data or use of other resources, resulting in a 81.8–88.0% sensitivity and 95–97% specificity, notably more accurate than a logistic regression [39].

There have also been studies that developed ANNs which include different physiological data from pulse oximetry and features of the electrocardiogram. Pulse oximetry data incorporated in an ANN proved to be a valuable screening tool with a high negative predictive value of 97.61%, utilizing a cut off of AHI ≥ 15 [40]. Another ANN solely utilized the blood oxygen saturation signal (SpO2) to predict AHI and oxygen desaturation index (ODI), classifying 90.4% and 94.4% of patient into the correct severity category based on estimated AHI and ODI respectively [41]. Physiological data from a single-lead ECG has also been utilized within an ANN to create an accessible, fast, portable method for diagnosis of sleep apnea [42].

Implementation of AI into the diagnosis of OSA has the opportunity to minimize the cost associated with diagnosis. A cost analysis study demonstrated that the ANN (OSUNet) was the most cost effective as a prediction tool compared to HSAT and other screening questionnaires [43].

Although the scope of this article is primarily focused on AI application of OSA diagnosis, there is also opportunity for this technology to be applied to optimizing treatment between surgery, use of OA, CPAP, or MT. An ANN has been utilized in achieving optimal CPAP pressure and decrease CPAP titration failure [44]. A machine learning-based intelligent monitoring system has also been shown as a cost-efficient method to increase CPAP compliance, with an average increase of 1.14 h per day [45]. There is a potential role for AI and utilizing “Big Data” gathered from CPAP devices and PSG to develop personalized effective treatment for individuals with OSA, while accounting for patient preference in terms of management [46].

**Fig. 1:**
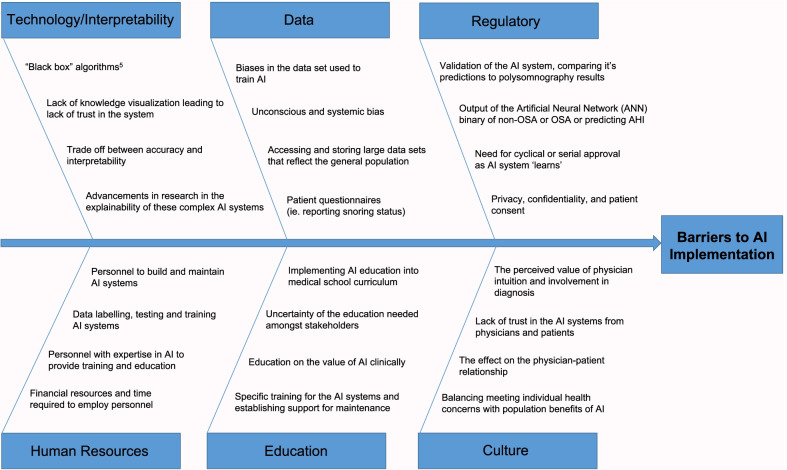
Barriers of artificial intelligence implementation in the diagnosis of obstructive sleep apnea

AI has the capacity to significantly benefit OSA diagnosis. Studies have shown the value of ANNs and the high specificity and sensitivity that is necessary for effective screening, notably much higher than based on subjective impressions of experienced sleep physicians, which detected OSA with a 60% sensitivity and 53% specificity [38]. The value of AI in the diagnosis of OSA is evident, yet there are still barriers that exist in the adopting of this tool clinically, outlined below and shown in Fig. 1.

### Barriers involved in AI implementation

#### Technology/interpretability

Neural networks allow for the consideration of the complexity of the relationship between variables contributing to risk of diagnosis of OSA, which allows for increased accuracy in detecting OSA. A limitation of this approach is “knowledge visualization,” as visualizing how the variables are interconnected and weighted within the network is virtually impossible [47]. Unlike logistical regressions, the relationships between the variables in ANNs are ambiguous and not easily understood [34]. ANNs are effectively “black boxes” as the variable inputs and the prediction made is known, but the pathways to reach the conclusion are not clearly understood as the computations cannot be elucidated clearly and do not display an “understanding of the structure of the reasoning.” [34] There is a balance between accuracy and algorithm transparency, where the most accurate models (deep learning and ANNs) are the least interpretable and the more intuitive models are less accurate (linear regression) [48]. In order for ANNs to be employed within OSA diagnosis, the importance of the accuracy of its predictions detecting OSA has to exceed the value of understanding the intricate pathways that compose the inner workings of the ANNs [49]. Research is being conducted within the field of computer science and explaining black-box algorithms, which will likely yield advancements that make black-box algorithms interpretable creating more trust in the technology, easing its implementation [50].

#### Data and biases

The reliability of the data utilized to train and validate AI systems is a significant obstacle for its implementation in the diagnosis of sleep apnea. Biases in the data set will create biases in the system and its predictive abilities, making this tool in diagnosis less useful and accurate [49]. In the development of a neural network designed to distinguish benign and malignant lesions, it was found that the algorithm was more likely to predict malignancy when a ruler was present on the image [51]. This example highlights the importance of the dataset utilized in the development of AI and the impact biases has on the clinical value of AI systems.

The access to the large data set necessary to develop AI tools and the long-term storage of this information creates an additional barrier for its implementation. Most AI systems developed by researchers utilize smaller data sets that do not accurately reflect the general population. The model developed by Kirby et al. used data from a population of patients who have been referred to a sleep clinic and therefore had a high prevalence of OSA (69%) [33]. Another ANN was also created using data from a population more likely to have OSA, not including those who were younger, had lower BMIs and did not report snoring [38]. In order to utilize these models within the general public as a screening tool and avoid unnecessary PSG, it would have to be tested with data with a lower prevalence of OSA [33]. While a general population dataset was utilized in the development and testing of the ANN studied by Li et al., further validation in other populations would be required for screening in high risk groups [40].

Systemic biases can impact the data source or not properly consider the impact of race, ethnicity, gender and other social determinants of health [50]. Although the prevalence of OSA is relatively similar across North America, Europe, Australia and Asia, the burden of the disease is higher in subsets of the population, including overweight and obese people, racial minority groups and older people [52]. It is important to consider how systemic and unconscious bias can affect data sources and selection and influence AI systems, especially in the context of OSA which has risk factors often found in marginalized groups. Incorporating local data in the training and testing of AI systems allows the screening tool to align with the “context-specific patterns of care.” [53]

Many of the ANNs that have been developed to detect OSA have required data from patient questionnaires reporting on their snoring status [38]. The knowledge snoring status is often accessible through bed partner observations and may result in incomplete or unreliable data depending on the patient’s sleeping experience. This method of data collection may be a source of bias and impact the ability of the AI tool to accurately predict OSA.

#### Regulation

Regulatory bodies are utilized in prioritizing safety and establishing guidelines for new medical devices, products and drugs. Similarly, AI systems also require regulation in order to be implemented clinically within the diagnosis of sleep apnea. In order for AI tools to be adopted they must be validated for secure use and accuracy. An effective screening tool must have a high sensitivity, as to not miss significant cases of OSA which can have detrimental health effects when untreated [3]. Utilizing AI in the diagnosis of OSA would have clear benefits for patients but there must be regulation, validation and risk-assessment in place as well. AI tools, such as ANNs, are validated by comparing their prediction or outcome with the gold-standard in diagnosis, namely PSG. The studies that have developed ANNs, validated the networks with retrospective data and lacked prospective validation [33, 34, 38]. Unlike the two earliest ANNs developed for OSA diagnosis [33, 34], a more recent neural network developed with input from four variables obtained in the office setting, had a validation stage to determine network’s architecture [38]. The size of the data set is also a limitation in the development of AI tools, and it is predicted that accuracy and performance will improve with exposure to more data. Since AI systems change and ‘learn’ there is also a question of whether these systems require serial cycles of approval [54]. It is important to establish regulation that protects patient privacy and ensures accuracy while allowing for the technology to learn so the benefit of AI can be implemented clinically.

Implementing AI into diagnosis and decision making is difficult when the “ground truth is subjective,” for example in Gleason grading of prostate cancer [55]. The diagnosis of OSA is less reliant on the interpretation, observation and annotation by physicians, as it is based on clinical symptoms and elevated AHI [56]. However scoring criteria and cut-offs for AHI events often vary across labs and studies [34]. One ANN that was discussed above combats this issue by utilizing AHI as the output in order to demonstrate severity of OSA rather than the binary of OSA or non-OSA [34]. In order to utilize AI tools effectively in the diagnosis of OSA in the public, there has to be regulation of the variables utilized and validation across multiple sleep labs and physicians.

#### Privacy and confidentiality

In order for the implementation of AI in the diagnosis of sleep apnea in the general population, the data set employed must be large and representative of the general population which is difficult to do in a research setting. The primary limitation in accessing larger data sets is patient privacy and data sharing [50]. The use of AI in healthcare challenges the ideology of privacy and confidentiality as “manageable, predictable and controllable,” as patients data is digitalized and utilized in complex algorithms [57]. New guidelines to ensure privacy and ethical data management are necessary for this novel context. There have been strides to ensure the appropriate regulation to protect individuals’ privacy while benefiting from AI, in utilizing public consultation to develop legislative changes to the Personal Information Protection and Electronic Documents Act (PIPEDA) [58].

In development of AI intended for OSA diagnosis, data is often collected from questionnaires, patient charts, and physiological testing. Patient consent is an important piece of data collection, and a study suggests that particularly for AI applications providing consent should be a dynamic process rather than a singular event as the evolution of the research and the technology develops [57]. Obtaining consent from patients often involves releasing their ownership of the information but the understanding of who owns and is responsible the data can be obscured as data can be transferred or bought by commercial companies [59]. Anonymized open-access data may address the question of ownership and privacy, but as data sets proliferate, the ability to combine data sets re-identification may be possible [60]. While the accuracy of AI tools increases with more data exposure, it is critical to consider the implications of digitalization of information on privacy and develop guidelines that protect patient information.

#### Human resources

The development of AI systems requires knowledgeable personnel “to build, maintain and improve AI systems.” [50] Tasks such as data labeling, training and testing the AI system all require resources, in respect to time, finances and the expertise of the personnel [50]. Establishing training programs for healthcare workers in AI and the specific algorithms additionally requires human resources. The time needed for education as well as the tasks of maintaining the system are important to consider as well. However, the amount of resources required to implement AI in the setting of OSA diagnosis is comparable to and potentially less than PSG testing long term, which requires the expertise of a polysomnographic technician and additional laboratory resources.

#### Environment/culture

The current medical culture opposes the idea that a computer can be involved in diagnosis and intimately entangled in crucial decisions regarding patient health. Moreover, physician intuition is often valued higher than evidence-based technological approaches [49]. However, in the diagnosis of OSA, physicians relying on their subjective intuition detects OSA with a specificity of 65% and sensitivity of 60%, drastically less than the ANNs that have been developed and discussed above [38]. While the logic AI offers is superior and demonstrates high accuracy, human intuition is not replaceable but can collaborate with the logical advances AI offers [61]. Of the stakeholders surveyed, in an analysis of the value of AI in laboratory medicine, a substantial proportion of those who reported AI as valuable were unsure of reasons supporting this stance and 19% did not perceive the adoption of AI to be valuable in their organization [62]. This highlights how education regarding AI and evidence based studies displaying it’s efficiency and value on a practical level are critical for shifting the paradigm regarding this emerging technology.

Additionally, attitudes of physicians contribute to a culture that is hesitant to embrace the adoption of AI into clinical practice. While healthcare workers confidently accept and utilize technology, such as PSG in diagnosing OSA and magnetic resonance imaging for other conditions, there is currently a lack of trust in AI technology [28]. Establishing validation and regulation of these systems may aid in building trust and increasing physicians comfort in adopting AI in diagnosis [55].

The patient–physician relationship also may be influenced by the adoption of AI clinically. If AI systems are responsible in medical decision making, the population concerns may take precedent over individuals [49]. In terms of OSA diagnosis, minimizing cost and conserving PSG resources has to be balanced with the priority of individual patient health concerns. The diagnostic accuracy of the AI system has to be high in order to protect patient’s health outcomes, particularly not missing severe diagnoses and streamlining their advancement towards treatment rather than further diagnostic testing with PSG. While AI can assist in diagnosis efficiency and accuracy, the physician’s role in the relationship remains important offering compassion and empathy [63].

#### Education

Education surrounding AI is important in shifting the culture and understanding of AI and increasing competency in its utilization,. Introducing AI education into the medical school curriculum is an important step, yet there are barriers including the dense curriculum, the knowledgeable personnel required to teach such skills and the uncertainty surrounding how AI will impact the future of heath care and the scope of healthcare workers responsibilities [50]. An article examining the challenges of building an AI competent workforce suggests basic literacy in AI could be developed alongside the medical school research and ethics curriculum, focusing on “outcome expectations and risk assessment.” [50]

In a study evaluating the value of AI in laboratory medicine, stakeholders were surveyed about what they felt was needed in order for AI to be adopted for diagnostic use [62]. While most participants responded unsure of what was needed, others highlighted the need for education. Specific training to the devices, AI courses and different support systems were suggested to help the adoption of AI into clinical practice [62]. The uncertainty reflected in the survey demonstrated the need for AI education and the development of an educational program which collaborates with stakeholders. The study also demonstrated that education on the value of AI itself is important for implementation of AI clinically and diagnostically [62]. Educating stakeholders and sharing the existing data that highlights the role and value of AI in the diagnosis of OSA would be important step in implementation. Education across different industries is also important and can aid in optimizing AI in the diagnostic and medical setting, with shared knowledge advancements and applications of AI in diverse settings.

## Conclusions

Artificial intelligence systems offer benefits to the diagnostic approach and treatment for OSA, improving accuracy and utilization of resources. The barriers identified were categorized into different themes including technology, data, regulation, human resources, education, and culture. Many of these challenges are ubiquitous across artificial intelligence implementation in any medical diagnostic setting. Future research directions include developing solutions to the barriers presented in this project.

## Data Availability

Data sharing is not applicable to this article as no datasets were generated or analysed. All material included in the review was accessed through PubMed and outlined in the references section.

## Abbreviations

AHI: Apnea Hypopnea Index
AI: Artificial intelligence
ANN: Artificial neural network
BMI: Body Mass Index
CPAP: Continuous positive airway pressure
CT: Computerized tomography
ECG: Electrocardiogram
EEG: Electroencephalogram
EMG: Electromyography
EOG: Electrooculogram
GRNN: Generalized regression neural network
HSAT: Home sleep apnea testing
MLP: Multilayer perceptron
MT: Myofunctional therapy
OA: Oral appliance
ODI: Oxygen desaturation index
OSA: Obstructive sleep apnea
OSUNet: Ohio State University Network
PSG: Polysomnography
PIPEDA: Personal Information Protection and Electronic Documents Act
STOP-Bang: Snoring, tiredness, observed apnea, high blood pressure-BMI, age, neck circumference, gender
